# Cell aggregation mediated by *ACE2* deletion in *Candida auris* modulates fungal colonization and host immune responses in the skin

**DOI:** 10.1128/msphere.00734-24

**Published:** 2024-10-30

**Authors:** Abishek Balakumar, Abigail Cox, Shankar Thangamani

**Affiliations:** 1 Department of Comparative Pathobiology, College of Veterinary Medicine, Purdue University, West Lafayette, Indiana, USA; 2 Purdue Institute for Immunology, Inflammation and Infectious Diseases (PI4D), West Lafayette, Indiana, USA; University of Guelph, Guelph, Ontario, Canada

**Keywords:** *Candida auris*, colonization, skin immune response, cell aggregation

## Abstract

*Candida auris* is an emerging multi-drug-resistant fungal pathogen that colonizes the skin and causes invasive infections in hospitalized patients. Multi-cellular aggregative phenotype is widely reported in the *C. auris* isolates, but its role in skin colonization and host immune response is not yet known. In this study, we generated aggregative phenotype by deleting the *ACE2* gene in *C. auris* and determined the fungal colonization and host immune response using an intradermal mouse model of *C. auris* skin infection. Our results indicate that mice infected with *ace2*Δ strain had significantly lower fungal load after 3 and 14 days post-infections compared to the non-aggregative wild-type and the *ACE2* reintegrated strain. The colonization of *ace2*Δ is associated with increased recruitment of CD11b^+^ Ly6G^+^ neutrophils and decreased accumulation of CD11b^+^ Ly6 C^hi^ inflammatory monocytes and CD11b^+^ MHCII^+^ CD64^+^ macrophages. Furthermore, Th17 cells and type 3 innate lymphoid cells (ILCs) were significantly increased in the skin tissue of *ace2*Δ infected mice. Our findings suggest that aggregative phenotype mediated by *ACE2* deletion in *C. auris* induces potent neutrophil and IL-17-mediated immune response and reduces fungal colonization in the skin.

**IMPORTANCE:**

*C. auris* is a rapidly emerging fungal pathogen that can colonize hospitalized patients, especially in skin tissue, and cause invasive infections. *C. auris* isolates exhibit morphological heterogeneity, and the multicellular aggregative phenotype of *C. auris* is reported frequently in clinical settings. Understanding the role of fungal morphotypes in colonization, persistence, and immune response in the skin microenvironment will have potential applications in clinical diagnosis and novel preventive and therapeutic measures. Here, we utilized the murine model of intradermal infection and determined that the aggregative phenotype of *C. auris* as the result of *ACE2* gene deletion elicits potential innate and adaptive immune responses in mice. These observations will help explain the differences in the skin colonization and immune responses of the aggregative morphotype of *C. auris* and open the door to developing novel antifungal therapeutics.

## INTRODUCTION

*Candida auris*, a rapidly emerging multi-drug resistant fungal pathogen, can cause invasive infections in humans (1). The U.S. Centers for Disease Control and Prevention (CDC) and the World Health Organization (WHO) classified *C. auris* as an urgent threat and critical priority pathogen (2, 3). Unlike other *Candida* species, such as *Candida albicans,* that colonize the intestine, *C. auris* has a unique ability to colonize and persist in human and murine skin long term, resulting in nosocomial transmission and outbreaks of systemic infections (4–8). Furthermore, our recent findings suggest that skin immune responses to *C. auris* are different from *C. albicans* (5). However, the factors that regulate *C. auris* skin colonization and immune response are poorly understood.

Clinical isolates of *C. auris* can exhibit different morphotypes, such as yeast, filamentous, and aggregative forms (9–13). Emerging evidence shows that aggregative phenotype affects the virulence, antifungal susceptibility, and biofilm formation of *C. auris* (11, 14, 15). The antifungal susceptibility of clinical isolates of aggregative *C. auris* shows reduced susceptibility to fluconazole (14). On the other hand, the exposure to triazoles and echinocandins also induced cell aggregation in *C. auris* clinical isolates of the South Asian clade, which are non-aggregative (16).

Cell aggregation in *C. auris* is a complex phenomenon, and more than one mechanism has been proven responsible (17). Broadly, *C. auris* can undergo adhesin-dependent and -independent aggregation (18). Recently, the forward and reverse genetic screening in *C. auris* identified several morphogenic mutants with irregular colony morphologies. Among them, insertional mutation in the *ACE2* gene resulted in an aggregative phenotype. *ACE2* is a zinc finger transcription factor expressed during the G_2_ phase and controls the expression of chitinase *CTS1*, which is required for septal degradation and cell separation (9). Without the *ACE2* transcript, daughter cells fail to separate after budding; the cells remain clumps or aggregates resistant to physical stress and disruption (14). Recent evidence suggests that the clinical isolates of *C. auris* with rough morphological colonies were reported to harbor single nucleotide polymorphism (SNP) at the *ACE2* locus (19). Furthermore, the experimental host-driven evolution of *C. auris* in the murine model of systemic infection identified multicellular aggregative morphologies with point mutations at several gene loci (20). Among them, suppression of the *ACE2* locus due to point mutations or loss of ORF region was reported during *C. auris* adaptive evolution in the host. Although these studies highlighted the importance of the *ACE2* gene in *C. auris* morphogenesis, its role in skin colonization and immune response is not yet known.

In this study, we examined the murine skin colonization and host immune response in the aggregative phenotype of *C. auris* due to the loss of the *ACE2* locus. We generated *ace2*Δ by deleting the *ACE2* gene in the South Asian AR0387 strain, a non-aggregative wild-type (WT) strain of *C. auris*. Using the intradermal murine skin infection model, we examined the colonization and skin immune response of aggregative (*ace2*Δ) and non-aggregative (WT) forms of *C. auris* AR0387. Our results suggest that fungal colonization and skin immune response of aggregative form due to *ACE2* deletion are distinct from the non-aggregative morphotype of *C. auris*.

## RESULTS

### *In vitro* growth and *in vivo* skin colonization regulated by *ACE2* deletion in *C. auris*

**Fig 1:**
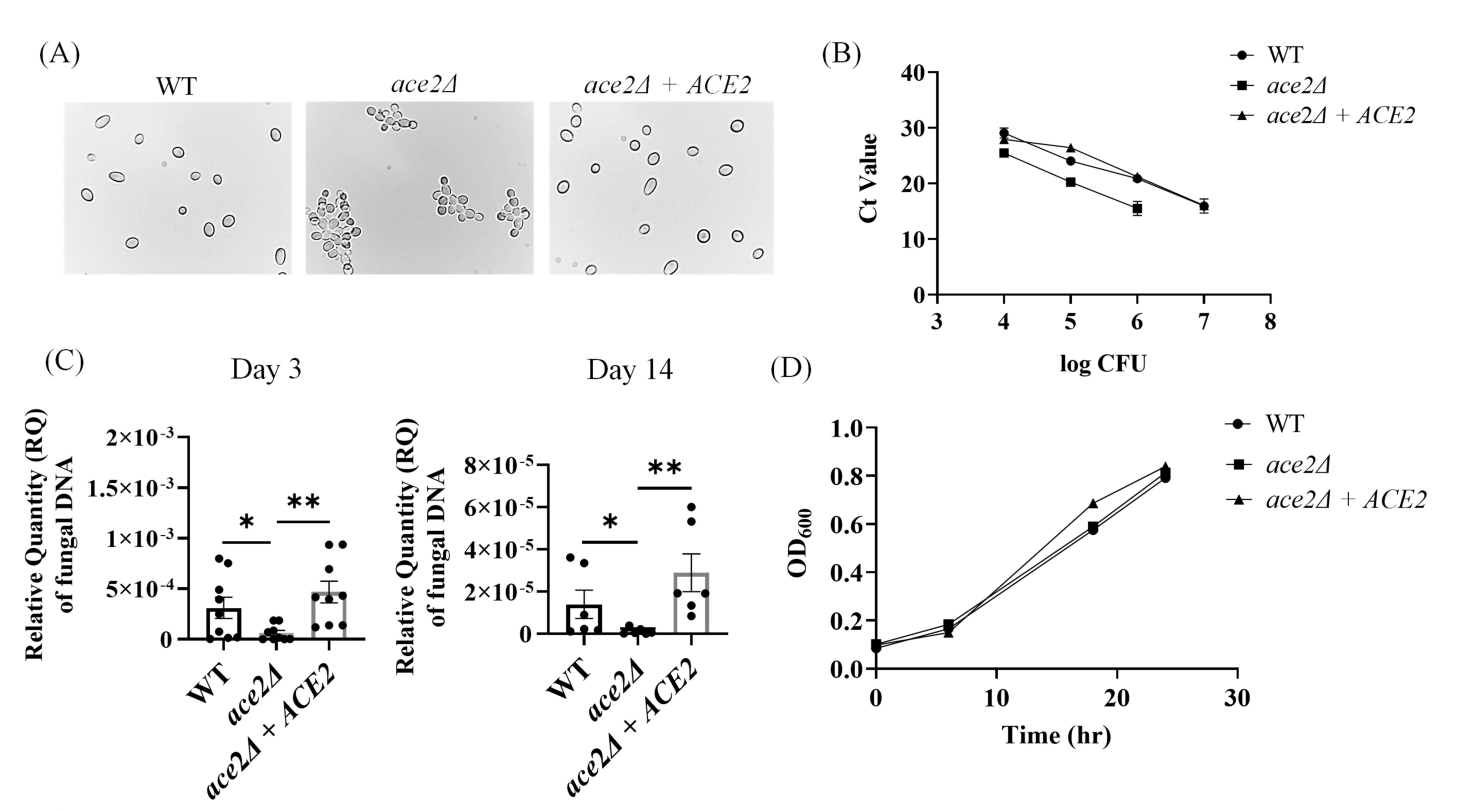
*In vitro* growth and *in vivo* skin colonization regulated by *ACE2* deletion in *C. auris*. (**A**) Microscopy of *C. auris* AR0387 (WT) *ace2*Δ and *ace2*Δ *+ ACE2* strains in differential interference contrast. Cells were grown in YPD at 30°C for 16 h, and images were taken under 400 × magnification. Bars, 100 µm. (**B**) Standard curve of the 10^7^, 10^6^, 10^5^, and 10^4^ fungal cells of WT, *ace2*Δ, and *ace2*Δ *+ ACE2* strains quantified using qPCR. The *x*-axis represents the colony forming units, and the *y*-axis represents the cycle threshold values. (**C**) Fungal load of murine skin infected with either WT or *ace2*Δ or *ace2*Δ *+ ACE2* after days 3 and 14. The bar graph represents the relative quantity of fungal DNA in murine skin tissue quantified in 25 grams of tissue weight in the WT, *ace2*Δ, and *ace2*Δ *+ ACE2* infected groups compared using Mann-Whitney U test, *P ≤ 0.05, **P ≤ 0.01. (**D**) Growth curve of *C. auris* WT*, ace2*Δ*, and ace2*Δ *+ ACE2* strains after 0 h, 6 h, 18 h*,* and 24 h. The strains were grown in RPMI-MOPS at 30^0^ C. The growth curve of the three groups was analyzed using one-way ANOVA, and there was no statistical significance between the groups.

We used CRISPR-Cas9 to delete the *ACE2* gene and reintegrate the gene back into the *ace2Δ* as described previously (21). *ace2*Δ and *ace2*Δ *+ ACE2* were confirmed by PCR and Sanger sequencing (Table S1). The *ACE2* deletion in the *C. auris* AR0387 strain results in a multicellular aggregative phenotype consistent with previous findings (Fig. 1A) (9). Due to the aggregative nature of the *ace2*Δ, CFU may not correspond to the individual fungal cells. Therefore, we employed quantification of the fungal DNA of *C. auris* using qPCR and determined that the fungal DNA copy number of *ace2*Δ does not correlate with the CFU of the wild type (WT) and *ace2*Δ *+ ACE2* (Fig. 1B). Hence, we counted the cells of *ace2*Δ and WT. The CFU to cell number ratio was determined by qPCR amplification of fungal DNA copy number compared with WT *C. auris* 0387 to establish the infection dose for *in vivo* murine infection (Table S2). Next, we examined the skin colonization of mice infected with *ace2*Δ and compared it with mice infected with the WT strain after 3 and 14 days post-infection. Due to the aggregative nature of the *ace2*Δ, the fungal DNA was quantified from the infected mice’s skin tissues to assess the fungal load. Our results indicate that mice infected with *ace2*Δ had significantly lower fungal load in the skin tissue after 3 and 14 days post-infection compared with mice infected with WT *C. auris* or *ace2*Δ *+ ACE2* strain (Fig. 1C). Taken together, our findings suggest that the mice infected with *ace2*Δ had lower skin fungal load compared with mice infected with non-aggregative WT *C. auris* strain. The reintegration of *ACE2* in the *ace2*Δ strain restored the difference in the fungal burden in the mice. Mice infected with *ace2*Δ *+ ACE2* complemented strain had increased fungal load similar to mice infected with the WT *C. auris* strain (Fig. 1C). Next, we determined the *in vitro* growth of the *ace2*Δ to assess if the difference in the skin fungal burden in the mice was due to the growth defects of the *ACE2* deletion in *C. auris*. We observed that there was no difference in the growth curve of the *ace2*Δ compared with the WT *C. auris* or *ace2*Δ *+ ACE2* strain (Fig. 1D). Taken together, our findings suggest that aggregative phenotype mediated by *ACE2* deletion in *C. auris* reduces fungal colonization in the skin.

### Mice infected with *ace2*Δ strain with aggregative phenotype induce a potent neutrophil response

**Fig 2:**
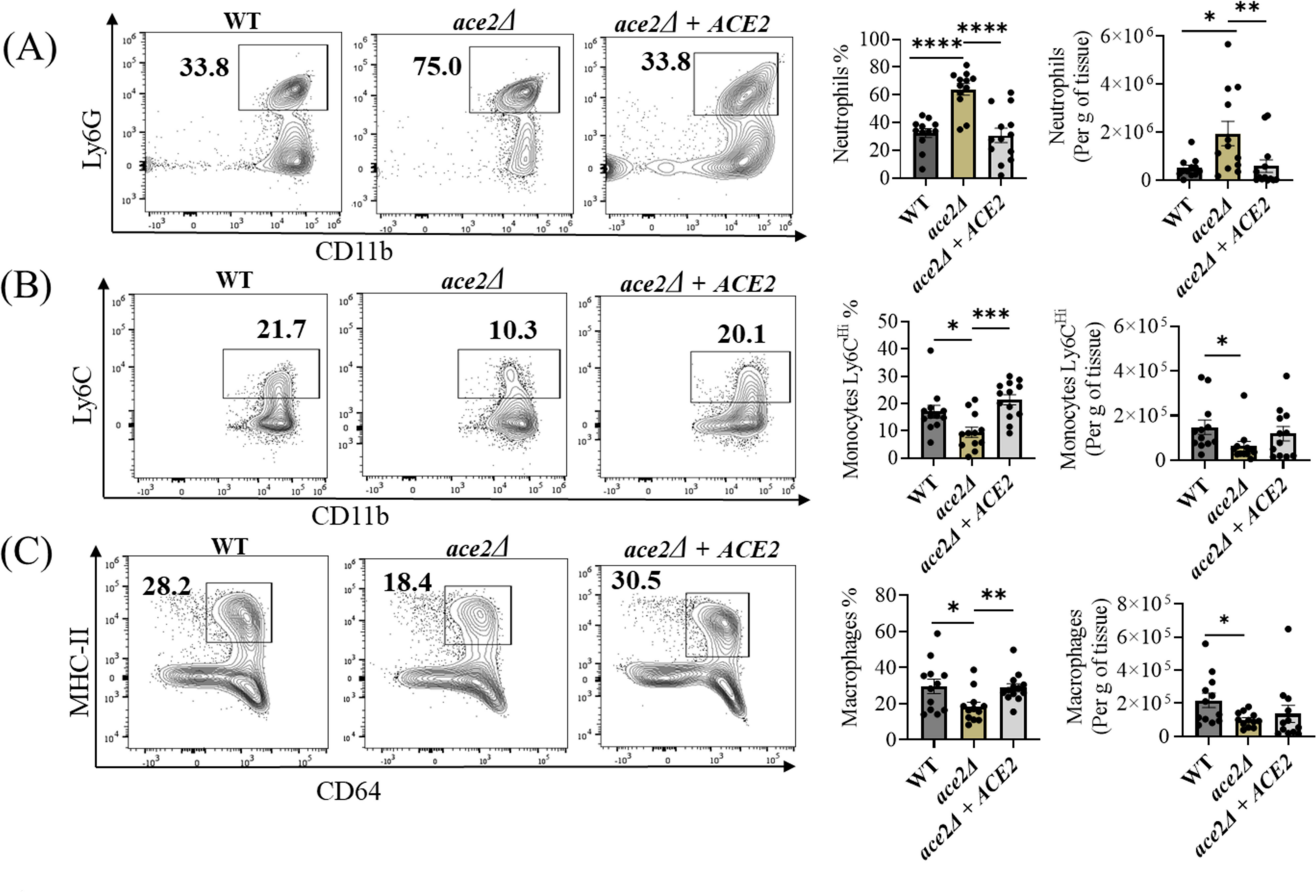
The *ace2*Δ aggregative strain induces a potent neutrophil response in the murine skin after 3 days post-infection. Representative flow plots, percentage, and absolute number of (**A**) CD11b^+^ Ly6G^+^ neutrophils, (**B**) CD11b^+^ Ly6C^Hi^ monocytes, and (**C**) CD11b^+^ MHC-II^+^ CD64^+^ macrophages in *C. auris* WT or *ace2*Δ infected murine skin after 3 days post-infection. Data are represented in a bar graph as mean ± SEM for each group obtained from 12 infected mice compared using the Mann-Whitney U test. *P ≤ 0.05, **P ≤ 0.01, ***P ≤ 0.001, ****P ≤ 0.0001.

Hence, to understand if the difference in the *in vivo* skin fungal colonization between aggregative and non-aggregative strains is due to differences in skin immune responses, we examined the innate and adaptive immune cells of mice infected with WT, *ace2*Δ*,* and *ace2*Δ *+ ACE2* infected mice. Phagocytic cells such as neutrophils, monocytes, and macrophages play an important role in innate host defense against *C. auris* skin infection (5). We examined CD11b^+^ Ly6G^+^ neutrophils, CD11b^+^ MHCII^+^ CD64^+^ macrophages, and CD11b^+^ Ly6 C^hi^ inflammatory monocytes in the mice infected with WT, *ace2*Δ, (or) *ace2*Δ *+ ACE2* reintegrated strain of *C. auris* after 3 days post-infection. Phagocytic cells were stained and gated using flow cytometry as described previously (5). Mice infected with *ace2*Δ strain induce potent CD11b^+^ Ly6G^+^ neutrophil response compared with the WT or *ace2*Δ *+ ACE2* infected mice groups (Fig. 2A). Both the percentage and the absolute cell number of CD11b^+^ Ly6G^+^ neutrophil was significantly increased in the skin tissue of *ace2*Δ infected mice. On the other hand, the percentage and absolute cell number of CD11b^+^ Ly6 C^hi^ inflammatory monocytes and CD11b+ MHCII^+^ CD64^+^ macrophages were significantly decreased in the skin tissue of *ace2*Δ infected mice compared with WT or *ace2*Δ *+ ACE2* groups (Fig. 2C). Collectively, our findings suggest that percentage and absolute number of CD11b^+^ Ly6G^+^ neutrophils were significantly increased, whereas CD11b^+^ Ly6 C^hi^ inflammatory monocytes and CD11b^+^ MHCII^+^ CD64^+^ macrophages were decreased in the skin tissue of mice infected with *ace2*Δ strain in relative to the WT or *ace2*Δ *+ ACE2* infected groups.

### Mice infected with *ace2*Δ strain with aggregative phenotype induce potent type 3 ILCs

**Fig 3:**
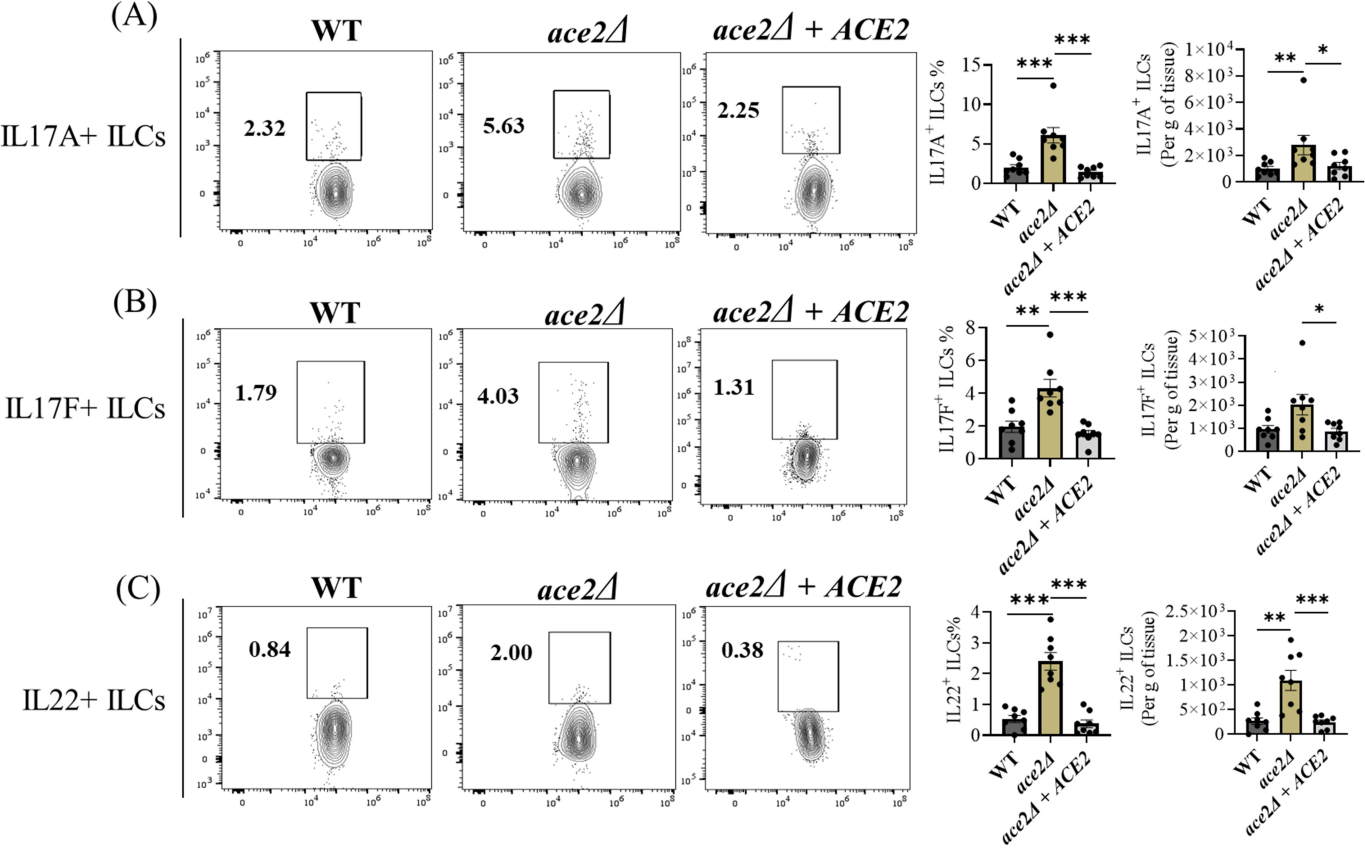
The *ace2*Δ aggregative strain induces a potent type 3 ILCs response in the murine skin after 14 days post-infection. Percentage and absolute number of (**A**) IL-17A^+^ ILCs, (**B**) IL-17F^+^ ILCs, and (**C**) IL-22^+^ ILCs in *C. auris* WT or *ace2*Δ infected mice skin tissue after 14 days post-infection. The samples from the different groups were processed independently for the intracellular staining and were gated with respect to their controls. Data are represented in a bar graph as mean ± SEM for each group obtained from eight infected mice compared using the Mann-Whitney U test. *P ≤ 0.05, **P ≤ 0.01, ***P ≤ 0.001.

Type 3 ILCs play a crucial role in barrier defense in the skin during fungal infection (22–24). We examined total ILCs, IL-17A^+^, IL-17F^+^, and IL-22 +ILCs in the skin tissue of mice of WT, *ace2*Δ, and *ace2*Δ *+ ACE2* infected groups. ILCs were strained and gated as described previously (5). The percentage of all three ILC subsets, IL17A+, IL17F+, and IL22 +ILCs, were significantly increased in *ace2*Δ mice relative to WT or *ace2*Δ *+ ACE2* groups (Fig. 3A through C). The absolute cell number of IL17A^+^, IL17F^+^, and IL22^+^ ILCs were significantly increased in *ace2*Δ mice skin tissue (Fig. 3A through C). Taken together, type 3 ILCs critical for host defense against fungal infection were significantly increased in the skin tissue of mice infected with *ace2*Δ relative to WT or *ace2*Δ *+ ACE2* groups.

### Mice infected with *ace2*Δ strain with aggregative phenotype induce potent IL-17-producing CD4+ T helper cells

**Fig 4:**
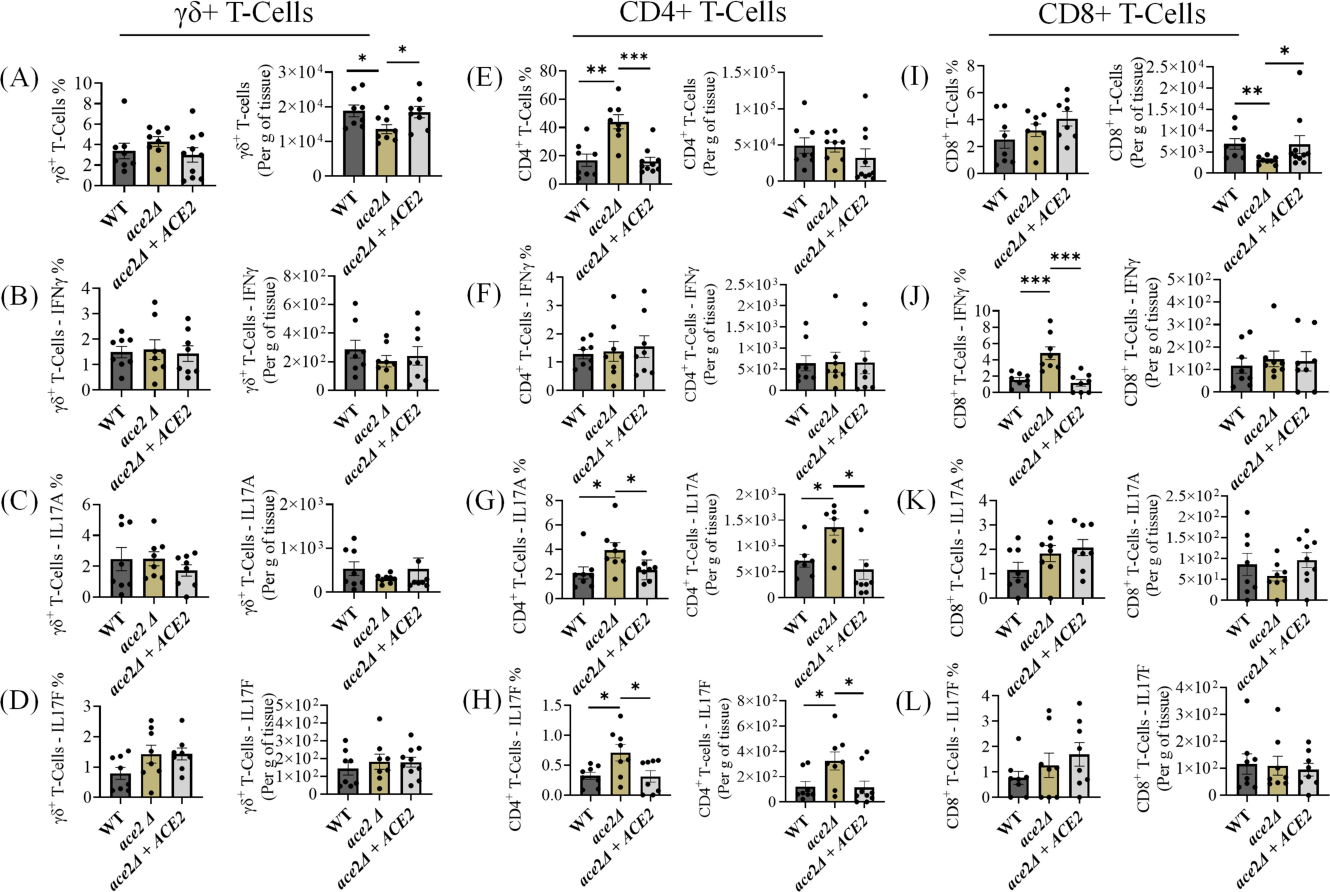
The *ace2*Δ aggregative strain induces a potent IL-17-producing CD4^+^ T helper response in the murine skin after 14 days post-infection. Percentage and absolute number of (**A-D**) total γδ^+^ cells, γδ^+^ IFN-γ^+^, γδ^+^ IL-17A^+^, and γδ^+^ IL-17F^+^. (**E-H**) Total CD4^+^ cells, CD4^+^ IFN-γ^+^, CD4^+^ IL-17A^+^, and CD4^+^ IL-17F^+^ T cells. (**I-L**) Total CD8^+^ cells, CD8^+^ IFN-γ^+^, CD8^+^ IL-17A^+^, and CD8^+^ IL-17F^+^ T cells in *C. auris* WT or *ace2*Δ infected mice skin tissue after 14 days post-infection. Data are represented in a bar graph as mean ± SEM for each group obtained from 8 to 10 infected mice compared using the Mann-Whitney U test. *P ≤ 0.05, **P ≤ 0.01, ***P ≤ 0.001.

IL17 is vital for the host defense against fungal infections, especially *Candida* spp. (4, 25, 26). We examined IFNγ-, IL17A-, and IL17F-producing T cell subsets, including γδ+, CD4+, and CD8^+^ T cells, after 14-days post-infection. T cells were stained and gated as described previously (5). The absolute number of total γδ^+^ T cells was significantly lower in the skin tissue of *ace2*Δ infected mice compared with the WT and *ace2*Δ *+ ACE2* groups (Fig. 4A). However, the percentage and absolute number of IFNγ, IL17A, and IL17F producing γδ^+^ T-cell population remains unchanged in *ace2*Δ infected mice (Fig. 4B through D) (Fig. S2 and S3). The percentage of total CD4^+^ T cells was significantly increased in the skin tissue of mice infected with *ace2*Δ compared with the WT and *ace2*Δ *+ ACE2* groups. However, the absolute number of total CD4^+^ T cells remains unchanged (Fig. 4E). There were no changes in the percentage and absolute number of CD4^+^IFNγ^+^ cells among the *ace2*Δ infected groups compared with the WT and *ace2*Δ *+ ACE2* groups (Fig. 4F) (Fig. S2). However, the percentage and absolute numbers of CD4^+^IL17A^+^ and CD4^+^IL17F^+^ cells were significantly increased in the skin tissue of mice infected with *ace2*Δ compared with WT or *ace2*Δ *+ ACE2* infected mice (Fig. 4G and H) (Fig. S3). The absolute number, but not the percentage of total CD8^+^ T cells, was significantly decreased in the skin tissue of mice infected with *ace2*Δ (Fig. 4I). Similarly, the percentage of IFNγ-producing CD8^+^ T-cells was increased dramatically in the skin tissue of mice infected with *ace2*Δ compared with the WT and *ace2*Δ *+ ACE2* groups (Fig. 4J) (Fig. S2). On the other hand, there were no changes in the percentage and absolute number of IL17A^+^ and IL17F^+^ producing CD8^+^ T-cell population in *ace2*Δ-infected skin tissue (Fig. 4K and L) (Fig. S3). Taken together, IL-17-producing CD4^+^ T cells, which are important for host defense against *C. auris* skin infection, were significantly increased in the skin tissue of mice infected with *ace2*Δ relative to WT or *ace2*Δ *+ ACE2* infected groups.

## DISCUSSION

The aggregative morphotype was recently reported in the clinical isolates of *C. auris* (14–16, 19). Since *C. auris* preferentially colonizes human skin, understanding the colonization, persistence, and skin immune response to different morphotypes of *C. auris* is critical to gain insights into the pathogenesis of this emerging fungal pathogen. The inherent difficulty in conducting *in vivo* studies using aggregative forms of *C. auris* is enumerating the fungal cells for infection and quantifying the fungal load recovered from tissues or organs. Physical disruption of aggregation by sonication was employed previously to enumerate the aggregative forms of *C. auris* (20). In this study, we adapted quantification of fungal DNA copy number to access the *in vivo* fungal load from mice skin tissues, since the traditional CFU plating may not accurately represent fungal burden. We observed that the mice infected with the *ace2*Δ exhibited significantly lower fungal burden at early time point, 3 days post-infection, and at late time point, 14 days post-infection. The *ACE2* deletion in *C. auris* did not confer growth defects. Hence, the difference in the *in vivo* fungal burden of *ace2*Δ aggregative form in the murine skin tissue is not due to the difference in the growth physiology. Our findings suggest that the difference in the skin fungal burden directed us to examine the skin immune response elicited by the *ace2*Δ aggregative form.

Next, we observed that neutrophils, inflammatory monocytes, and macrophages were distinct in the skin tissue of mice infected with *ace2*Δ strain. Neutrophils are crucial for fungal defense (27, 28). Recent evidence suggests that neutrophils are less efficient in recognizing and killing *C. auris* (29) due to the presence of a dense mannan layer (30) and recruit significantly fewer neutrophils than the *C. albicans* in the murine infection (5, 31). Surprisingly, we observed a significantly increased accumulation of CD11b^+^ Ly6G^+^ neutrophils in the skin tissue of mice infected with *ace2*Δ compared with mice infected with non-aggregative phenotypes. The increased neutrophil recruitment of *ace2*Δ in the murine skin may be due to changes in morphology or in the fungal cell wall pathogen-associated molecular patterns (PAMPs), especially chitin, which was previously characterized in *C. auris* (9). The increased neutrophil response also correlates with the lower fungal burden recovered from the skin tissue of the *ace2*Δ-infected mice.

Macrophages play a critical role in innate defense against *Candida*; its role in *C. auris* has been recently explored (32). Recent evidence suggests that *C. auris* replicates within macrophages by bypassing the *NLRP3* pathway (33, 34). Our findings indicate that mice infected with *ace2*Δ induce a less potent macrophage response than non-aggregative WT groups. Similarly, the *ace2*Δ infected mice recruited significantly low CD11b^+^ Ly6C^hi^ inflammatory monocytes. Although the role of macrophages and monocytes in skin defense against *C. auris* is rudimentary, the morphology and cell wall PAMPs of *C. auris* have a potential role in the innate phagocytic response. A recent *in vitro* study on co-culturing of the aggregative phenotype of *C. auris* with macrophage demonstrated that the macrophage does not recognize and clear the aggregates due to the morphology (18). Future studies to dissect how different morphotypes of *C. auris* are recognized by different phagocytic cells and their role in host defense are essential to understanding the host immune response to this emerging fungal pathogen.

The ILCs encompass the barrier immunity in the mucosal surfaces such as the intestine, skin, and lungs to prevent evading pathogens (35). The type 3 ILCs are distributed on the mucosal surface of the skin and promote defense against evading skin pathogens by promoting IL-17 and IL-22 responses (36). ILCs were known to promote IL-17 response in *C. auris* (4). The IL-17A, IL-17F, and IL-22-producing type 3 ILCs were significantly higher in the *ace2*Δ after 14 days post-infection. Although *C. auris* does not induce potent type 3 ILCs compared with C. albicans, the *ace2*Δ, on the other hand, induces significantly increased type 3 ILC response in the murine skin.

The IL-17-producing T cells, such as CD4^+^ T cells, CD8^+^ T cells, and γδ T cells, regulate *C. auris* skin colonization by promoting host defense in the skin (4). Previous findings from our laboratory identified that *C. auris* produces a significantly lower IL-17 response compared with *C. albicans* in the murine skin (5). Interestingly, the mice infected with *ace2*Δ elicited significantly increased CD4^+^ IL-17 response in the skin. We observed that a decrease in fungal load in the skin tissue of mice infected with *ace2Δ* after 14 days post-infection correlates with potent IL-17 response observed in the skin tissue of mice infected with *ace2*Δ relative to WT groups. However, future studies are required to understand how *C. auris* morphology drives specific T helper response, which is critical to developing novel therapeutic and vaccine strategies to control and treat this emerging fungal pathogen.

The RAM pathway in pathogenic fungi is the signaling network of protein kinases that regulate morphology and cellular functions. They are usually conserved between the species, and studying the fungal effectors that regulate cell cycle, cell separation, mating, polarized growth, maintenance of cell wall integrity, and stress signaling is important to understanding the biology and pathogenesis of the fungal species (37). The deletion of *ACE2* in *C. albicans* and *Saccharomyces cerevisiae* resulted in the attenuation of virulence, but in *Candida glabrata* provoked virulence (38, 39). Interestingly, the mice intravenously infected with host-evolved *C. auris* with suppression of *ACE2* locus did not survive after 5 days post-infection, denoting its virulence traits in *C. auris* (20). In our study, the lower fungal burden during the early and late time points of the murine skin infection with *C. auris ace2*Δ is accompanied by an enhanced innate and adaptive immune response. This may be potentially due to the increase in virulence or immunostimulatory property of *C. auris* when the fungal effector *ACE2* is deleted. Future studies to understand the downstream pathways of the fungal effectors that control the immune response and fungal persistence in the host are important to develop novel therapeutic strategies against this emerging fungal pathogen.

## MATERIALS AND METHODS

### Strains, media, and reagents

*C. auris* isolate AR0387 (South Asian) used in this study was procured from the Centers for Disease Control and Prevention’s Antibiotic Resistance Isolate Bank, USA. All the strains were stored at −80 in 25% glycerol stock and revived by plating in yeast extract-peptone-dextrose (YPD) agar. The strains were propagated in yeast extract-peptone-dextrose (YPD) broth before infection. Other reagents and antibodies used in the study for immune cell isolation and staining were described (5). Plasmids pCE27, pCE35, pCE41, and pCE38 were purchased from Addgene after signing the Biological Material Transfer Agreement. PhusionTM High Fidelity Polymerase (Thermo Scientific), 10 mM dNTPs (Thermo Scientific), restriction enzyme FastDigest *MssI* (ThermoScientific), and DreamTaq Green DNA Polymerase (5 U/µL) (Thermo Scientific, Cat. no. FEREP0712) used in the study were purchased from Thermofisher Scientific USA.

### *ACE2* deletion strain construction and reintegration

The *ACE2* deletion was constructed by CRISPR-mediated genome editing in *C. auris* using LEUpOUT system as described previously in (21). The oligonucleotides used in the study were provided in (Table S1). gRNA was designed for *ACE2* (B9J08_000468) using Benching by the following parameters: reference genome*—C. auris* B8441, guide design—single guide of 20 bp, PAM—NGG (SpCas9, 3′ side). The on-target score and off-target score cutoff were set to ≥50 to select gRNA. The gRNA cassette was constructed from pCE27 by amplification and subsequent PCR stitching of fragments A and B. In the amplification of fragment A, 20 bp gRNA “CTCAACGAAACCTCGTACAC” is incorporated by using a 60 bp forward primer with 20 bp overhangs on either side of gRNA, which has homology with the existing sequence in pCE27 to stitch. Fragment B is amplified from pCE27 plasmid using primers and then stitched with fragment A to construct fragment C for transformation. The CRISPR-Cas9 cassette was constructed from pCE35 plasmids by digesting with restriction enzyme *MssI* at 37°C for 10 min and inactivated enzyme at 65°C for 10 min before transformation. Donor DNA (dDNA) was constructed by amplifying and stitching the upstream and downstream of the *ACE2* gene intersecting at a 23 bp homology sequence CGAGACGAGTGCTCGACATGAGG (ADDTAG), replacing the *ACE2* ORF (Fig. S1A). dDNA fragments were amplified and stitched from the genomic DNA of *C. auris* AR0387. The construction of dDNA, gRNA cassette, and CRISPR-Cas9 cassette are shown in (Table S1). All the fragment constructs were transformed into *C. auris* AR0387 by the induction of competence using lithium acetate, single-stranded carrier DNA, and polyethylene glycol method followed by heat-shock at 44°C for 15 min for effective transformation. The transformants were selected on YPD agar plates supplemented with 300 mg nourseothricin (NAT) to select the cells carrying the transformed fragments and the desired edits. The deletion was verified using colony PCR by designing primers nesting the upstream and downstream of the *ACE2* ORF. The *ACE2* deletion was confirmed by agarose gel electrophoresis and Sanger sequencing (Fig. S1B) (Table S1). For the reintegration of *ACE2* gene in *ace2*Δ*,* the gRNA was designed at the PAM site in the ADDTAG sequence integrated at the *ACE2* ORF. The gRNA cassette and Cas9 cassette for *ACE2* complementation were constructed from pCE41 and pCE38 plasmids, and the transformants were selected in YPD supplemented with 600 mg of hygromycin (HYG) (Fig. S1C).

### Quantification of fungal DNA by qPCR

The fungal cells of WT *C. auris* 0387 or *ace2*Δ or *ace2*Δ *+ ACE2* strains were enumerated in the hemocytometer, and 10^7^, 10^6^, and 10^5^ cells were taken for fungal DNA extraction using the Yeast DNA Extraction Kit (Thermo Scientific) following manufacturer’s instructions. The fungal DNA was quantified using *C. auris*-specific primers, sense, AGAGTCGAGTGAGTCAAAAC, and antisense, CTCAACTCGGAATTTTTCATC, as described previously (40). The cycle threshold values of the fungal DNA amplified from 10^7^, 10^6^, and 10^5^ cells were plotted against the CFU determined to establish a standard curve for WT *C. auris* 0387, *ace2*Δ*,* and *ace2*Δ *+ ACE2* strains.

### Growth conditions and murine infection

Both the WT and *ace2*Δ *C. auris* strains were cultured in YPD broth at 30°C overnight with 250 rpm shaking. Then, the cells were centrifuged at 500 × *g* for 5 min and washed twice with 1× PBS. Then, the cells were re-suspended in 1× PBS, vortexed briefly, and optical density at 600 nm (OD_600_) was measured using a spectrophotometer (Synergy HT; Bio-Tek Instruments). Approximately 1.0 OD_600_ of culture was counted in a hemocytometer, and cells were diluted to ~10^7^ fungal cells per mL. Then, 100 µL of the diluted culture containing ~10^6^ fungal cells was used for murine infection. In *ace2*Δ, the cells were clumped together as aggregates, and a single colony would not represent a single cell (Fig. 1B). Hence, the cells were approximately counted in a hemocytometer, and the CFU to cell number ratio was determined by qPCR amplification of fungal DNA in comparison to WT *C. auris* 0387 to establish the infection dose in terms of fungal cells received by the mice (Table S2). Six to 8-week-old C57BL/6 J mice were used in this study. The murine infections performed in this study were followed as described previously (5). All the mice were infected intradermally with WT (or) *ace2*Δ strains of dose ~1–2 × 10^6^ fungal cells per mice.

### Skin fungal load determination by qPCR

Mice were euthanized after 3 and 14 DPI, and skin tissue was collected. Fungal DNA was isolated from the homogenate and quantified as described previously (41). Briefly, gDNA was extracted from 25 mg of tissue from each sample using Qiagen’s DNeasy blood and tissue kit. The tissue was mechanically homogenized in 1× PBS using an electric homogenizer (VWR, avantor, USA). Tissue homogenates were centrifuged at 17,000 × *g* for 5 min, and the supernatant was removed. Then, 180  µL of buffer ATL and 20  µL proteinase K were added to the pellet, and the fungal cell wall was disrupted by bead beating in BeadBug six homogenizer (Benchmark Scientific) for 10 cycles at a speed of 2,500. Then, the lysate was incubated at 56°C for 15  min, and the DNA was isolated following the manufacturer’s protocol. The eluted DNA from each sample was quantified using a NanoDrop 2000 c Spectrophotometer (Thermo Scientific). One hundred nanograms of input gDNA from the samples was used to quantify the presence of fungal DNA using qPCR. *C. auris*-specific gene primers were used for the quantification of fungal DNA copy numbers in the samples as described previously (40). The relative quantity of fungal DNA of *C. auris* was determined by normalizing the threshold cycle (CT) values of mouse GAPDH gene using the Δ*C_T_* method, since the fungal burden in skin tissue is proportional to the weight of the tissue.

### Determining fungal load in skin tissue

WT, *ace2*Δ*,* or *ace2*Δ *+ ACE2* infected mice were euthanized, and skin tissue weighing around 100–150 mg was collected. The tissue was mechanically homogenized in 1× PBS using an electric homogenizer (VWR, avantor, USA). The homogenate was serially diluted and spot-plated in a YPD agar plate supplemented with 100 µg/mL ampicillin, 50 µg/mL kanamycin, and 100 µg/mLstreptomycin and incubated at 37°C for 24 h to determine the fungal burden per gram of tissue as described previously (5).

### Growth assay

WT, *ace2*Δ*,* and *ace2*Δ *+ ACE2* strains were grown in YPD at 30°C at 250 RPM shaking for 16 h. The cells from the overnight culture were pelleted and washed with 1× PBS twice and enumerated in the hemocytometer. Then, ~10^6^ fungal cells per mL were seeded in RPMI-MOPS, and 100 µL was added to the flat-bottom 96-well microtiter plates and incubated at 30°C. The growth of the following strains was measured at 0 h, 6 h, 18 h, and 24 h by the OD_600_ using a microplate reader (Synergy H1; Bio-Tek Instruments).

### Immune cell isolation from mice skin tissue and antibody staining

Immune cells were isolated from the whole mice’s skin as described previously (5). Single-cell suspension was stained with respective surface and intracellular antibodies as described previously (5). After staining, the cells were washed and resuspended in cell staining buffer prior to sample acquisition. Then, the flow cytometric data were acquired in Attune NxT Flow Cytometer (Invitrogen, CA, USA) and analyzed using FlowJo (Eugene, OR, USA) as indicated before (5).

### Statistical analysis

All the statistical data analyses were performed using GraphPad Prism 9.4.1. Statistical significance was determined using the Mann-Whitney test to compare the mean between the two groups. Data were represented in mean ± standard error of the mean (SEM).

## SUPPLEMENTAL MATERIAL

Supplemental Material msphere.00734-24-s0001.docx Tables S1 and S2; Figures S1 to S3.

ASM does not own the copyrights to Supplemental Material that may be linked to, or accessed through, an article. The authors have granted ASM a non-exclusive, world-wide license to publish the Supplemental Material files. Please contact the corresponding author directly for reuse.
