# Supplementary material for: Cell aggregation mediated by ACE2 deletion in Candida auris modulates fungal colonization and host immune responses in the skin
Source: mSphere. 2024 Oct 30;9(11):e00734-24. doi: 10.1128/msphere.00734-24 (PMC11580408; doi:10.1128/msphere.00734-24)
Supplement: Supplemental Material — Tables S1 and S2; Figures S1 to S3. [file msphere.00734-24-s0001.docx]

**Table S1: Oligonucleotides used for CRISPR CAS9 mediated genome editing and sequence confirmation of *ACE2* deletion.**

| **Description** | Sequence |
| --- | --- |
| *ace2Δ* deletion gRNA | CTCAACGAAACCTCGTACAC |
| *ace2Δ* + *ACE2* complementation gRNA | CGAGACGAGTGCTCGACATG |
| Upstream forward primer for *ace2Δ* | CCTCACCCCAGTAGCTCTCA |
| Upstream reverse primer for *ace2Δ* | CCTCATGTCGAGCACTCGTCTCGAGCGGGCGCTGGTGAAATTT |
| Downstream forward primer for *ace2Δ* | CGAGACGAGTGCTCGACATGAGGCGTTGCATTTCTTTTTCCTT |
| Downstream reverse primer for *ace2Δ* | CGAAAAGTTCACATAATTTGCCCA |
| Colony PCR forward primer | GCAATCCTGGCCGTATACCA |
| Colony PCR reverse primer | AGCAATGGCGAATGTTACACG |
| Sequence of *ace2Δ* stain using colony PCR forward and reverse primers. | NNNNTTGNNNCTTNNNNNCNAGCNCCCCAAAGCTCCATTTCCCATTCAAGCTAATATTAGCTTTTCAAAATTTCACCAGCGCCCGCTCGAGACGAGTGCTCGACATGAGGCGTTGCATTTCTTTTTCCTTTTCAAAACATCTATTGTATTTTAAACCTTCCTTCTTATTTCATTTCAAAAGAAACTATTGGCATTTCATTCTTTCTCGTGTAACANNNCCATTGCNTNNN |
| Sequence of *ace2Δ* + *ACE2* stain using upstream forward and reverse primers. | >ACE2_Complement-ACE2_Fwd  NNNNNNNNNNNNNNNNNNTATCGCCCTCAGCAGCACATAAGTCATCTTTTGCATTTGTCCGACTTCCAGCACTTACGCCTATTATTTTTTTTCCTTTCTATCATCCATCAAAGAAACTATACATTGCACTGCTGCTAGAGTGGCTGCAAAATCCAATCTAGAAAAGCTACACCCTGCTAGCAATCCTGGCCGTATACCATATCTTCCTATAAGTGATTGCAGCTTCAACTCCTAGCAACCCCAAAGCTCCATTTCCCATTCAAGCTAATATTAGCTTTTCAAAATTTCACCAGCGCCCGCTATGGATCCCTACTGGGAGGACCTTGACTTCTCAACGAAACCTCGTACACCGGCACCAGAGGAAGAGTTCAGCCAGTACTTTCTGGACTACAAAAACTTC  GATAACCTCTTCAATGAGGCATTGACTACTCTTCAGGATTTGGACGTGCCTTCAGGGCCACCACAGCCTGCCCAAGTAGCAGCTTTCCAATCTCCTTTCAGACATGCAAAGAAACCTAGTGGAACTGCCATCTTTGGTTTTGCTGACCACAGCAGAGAGCTTTCGATAAACGGCATGACCAATGAATATCTTAAACACCAAAGGGCGCCCTCAGAGGCGTCGTCTTATATTCTGCCGGGGAAAATTGCAAGAAAAAAGGCTGCACCACCAATCTCAAACGATCACCTCGATTTCAACTTTTCGCAGCCATTGGNNGCCTGTANNNTTTTCAACTAAATGANATGANNGTATATGAAGGANAGCNCCTCCNNAGCNNANNANCNNNNN  >ACE2_Complement-ACE2_Rev_  NNNNNNNNNNNNNNNNNNNNCATGGCGANTGTTACACGAGAAAGAATGAAATGCCAATAGTTTCTTTTGAAATGAAATAAGAAGGAAGGTTTAAAATACAATAGATGTTTTGAAAAGGAAAAAGAAATGCAACGTCAACGTCTCTGGGCGGCGAGCCCCAAATCTTCGTGGAGGCGATTCGACGTAAGTCCGTCCAAAACAGCCTCCCCTCTCTTCAGCTTGATGGGACTTGGCTTTGACACTCCCTTTGTCGATCCACCCACTTCACTCTCGGAACTCTTCTCACAGATGCCCTTATTCAAATGTCTCTTGTAGCCTTCCACTCGAGTGAATTCTTTCCCACATTTACATCTGCAATGCTTTGCTACCAAGTGTGACTTTACATGTCTGTTGAGATCATGCTGACGTACAAAAGATTTGGGACAGTAGCTGCAAGTGAAGGGTCGGTCGCTTAGATGTGTTTGGATATGAGAACGAACGTTGTACCTTCTGGTAAACTTCTTACCGCATCCTTCATAAGTGCAAGTGAAGATCTTGTTCTCATCTGGTCCCTCCCAGTATCTATCTAGCTCCCCTGGCGGGAGCAAGGAAGTCTTTTTGATGACTCGTTTTGGAGAAAGGTCTTGAATGGCTTTGCGAACGTCTGTTGACTTTGAGTTGGGGGAAATGATCGGACTCCACTCTAGCTTAATCNNGTTTTTGCTAGNAGTGAATGGCGTAGCAGAATTCTTTAGTGGNGTAAGGACGATGGNATCATTCANN |
| qPCR; m-GAPDH – Forward primer | CTCCCACTCTTCCACCTTCG |
| qPCR; m-GAPDH – Reverse primer | GCCTCTCTTGCTCAGTGTCC |
| qPCR; *C. auris* specific primer – Forward | AGAGTCGAGTGAGTCAAAAC |
| qPCR; *C. auris* specific primer – Reverse | CTCAACTCGGAATTTTTCATC |

**Table S2: Fungal cells to colony forming unit ratio table for *C. auris ace2Δ.***

| **No. of Fungal Cells of *ace2Δ*** | **Colony Forming Unit (CFU) of *ace2Δ*** | **Ct Value of** ***ace2Δ*** | **Ct Value of WT *C. auris* 0387** |
| --- | --- | --- | --- |
| ~10^7^ | 1.7 × 10^6^ | 15.506 | 15.938 |
| ~10^6^ | 1.2 × 10^5^ | 20.258 | 20.885 |
| ~10^5^ | 1.1 × 10^4^ | 25.104 | 24.055 |

**
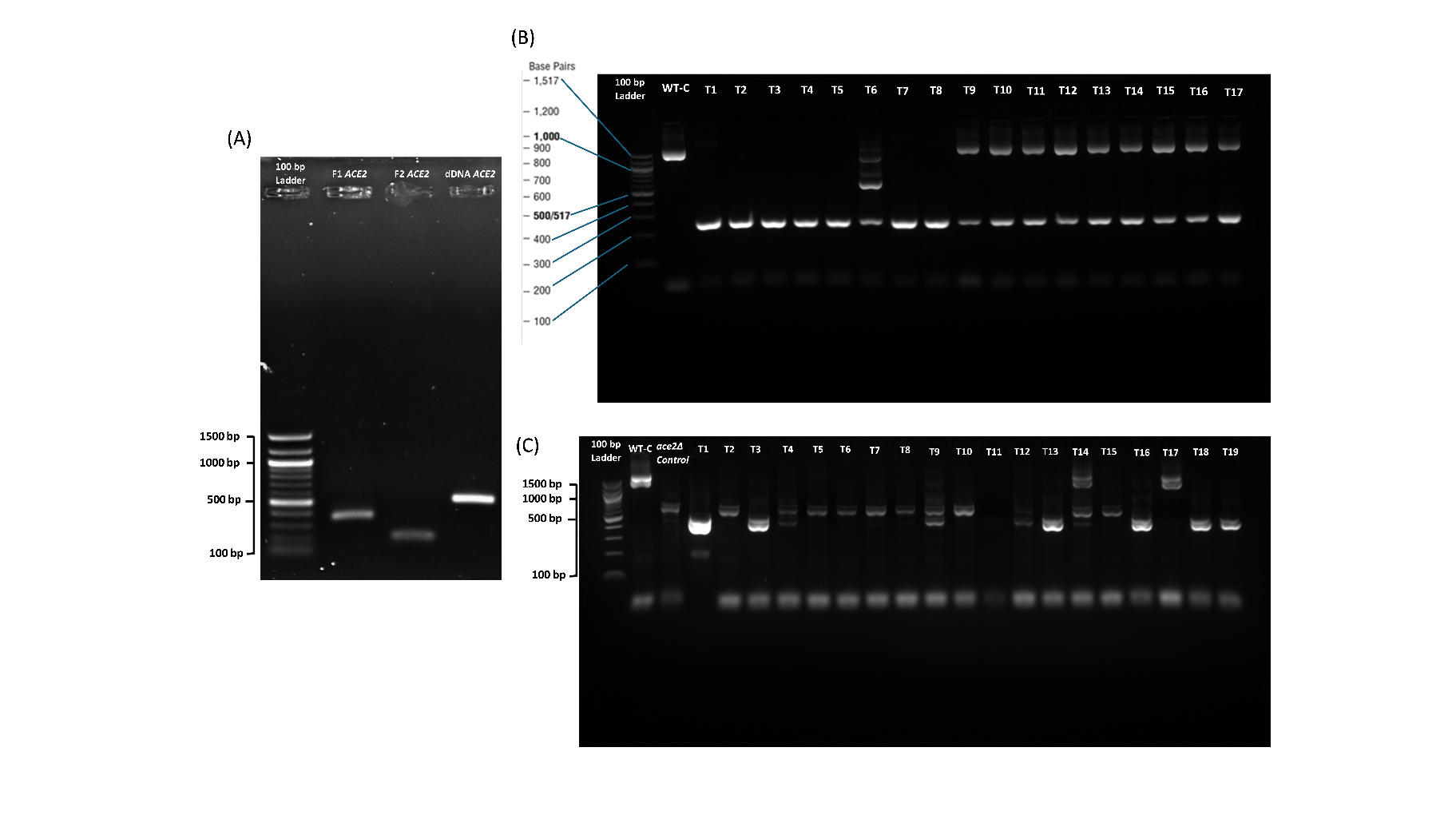
**

**Figure S1: Confirmation of *ACE2* deletion and reintegration in *C. auris* AR0387 strain by agarose gel electrophoresis.** (A) Construction of repair template for *ACE2* deletion. Amplification and stretching of F1 and F2 fragments. (B) Colony PCR screening of *ACE2* deletion in the transformants grown on the YPD + NAT 300 plate. (C) Colony PCR screening of *ACE2* reintegration in the transformants grown on the YPD + HYG 600 plate.

**
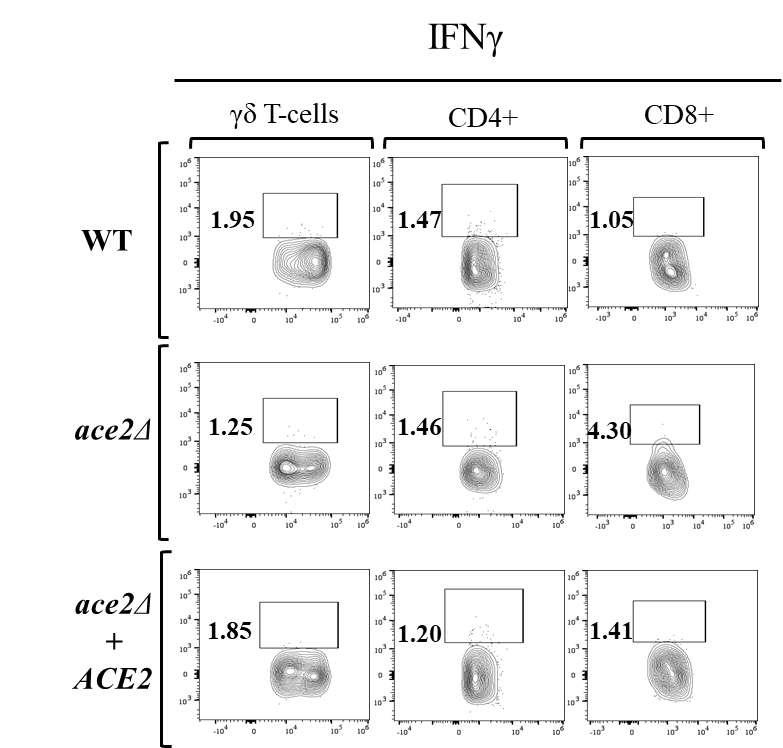
**

**Figure S2: Representative flow plots of IFN-γ producing γδ^+^, CD4^+^ and CD8^+^** **T-cell populations.** The flow plots represent the γδ^+^ IFNγ^+^, CD4^+^ IFNγ^+^, and CD8^+^ IFNγ^+^ population gated from the WT, *ace2Δ*, and *ace2Δ + ACE2* infected mice skin tissue.

**
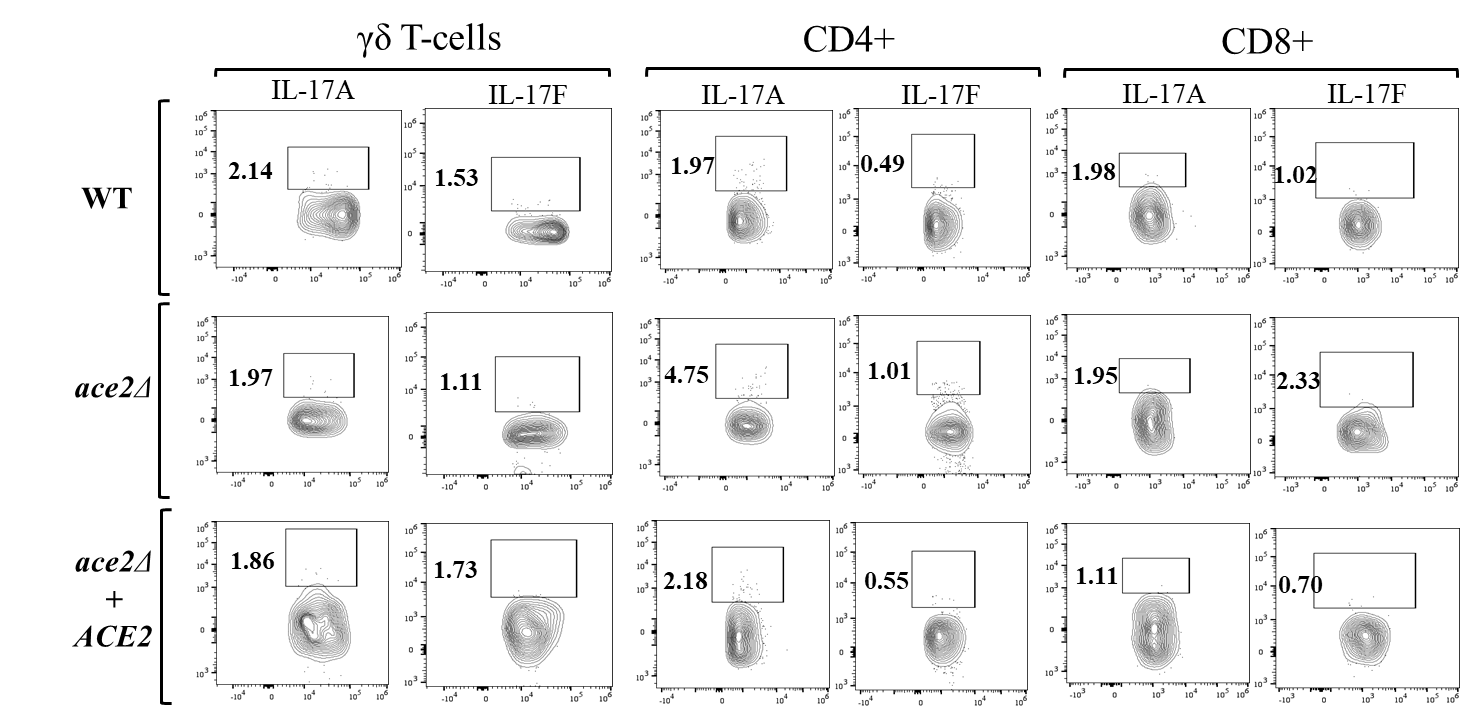
**

**Figure S3: Representative flow plots of IL-17 producing γδ^+^, CD4^+^ and CD8^+^** **T-cell populations.** The flow plots represent the γδ^+^ IL-17A^+^, γδ^+^ IL-17F^+^ CD4^+^ IL-17A^+^, CD4^+^ IL-17F^+^, CD8^+^ IL-17A^+^ and CD8^+^ IL-17F^+^ population gated from the WT, *ace2Δ*, and *ace2Δ + ACE2* infected mice skin tissue.
